# Brain insulin signaling and cognition: Possible links

**DOI:** 10.17179/excli2023-5841

**Published:** 2023-02-13

**Authors:** Habib Yaribeygi, Mina Maleki, Alexandra E. Butler, Tannaz Jamialahmadi, Amirhossein Sahebkar

**Affiliations:** 1 Research Center of Physiology, Semnan University of Medical Sciences, Semnan, Iran; 2 Urology and Nephrology Research Center, Shahid Beheshti University of Medical Sciences, Tehran, Iran; 3 Research Department, Royal College of Surgeons in Ireland, Bahrain, PO Box 15503, Adliya, Bahrain; 4 Applied Biomedical Research Center, Mashhad University of Medical Sciences, Mashhad, Iran; 5 Surgical Oncology Research Center, Mashhad University of Medical Sciences, Mashhad, Iran; 6 Biotechnology Research Center, Pharmaceutical Technology Institute, Mashhad University of Medical Sciences, Mashhad, Iran; 7 Department of Biotechnology, School of Pharmacy, Mashhad University of Medical Sciences, Mashhad, Iran

**Keywords:** diabetes mellitus, brain insulin signaling, cognition, senile plaque, mitochondria

## Abstract

Poor cognitive ability is a consequence of a wide variety of neurobehavioral disorders and is a growing health problem, especially among the elderly and patients with diabetes. The precise underlying cause of this complication is not well-defined. However, recent studies have highlighted the possible role of insulin hormone signaling in brain tissue. Insulin is a metabolic peptide integral to whole body energy homeostasis; it does, however, have extrametabolic impacts, such as upon neuronal circuits. Therefore, it has been suggested that insulin signaling may modify cognitive ability by yet unknown pathways. In the current review, we discuss the cognitive role of brain insulin signaling and consider the possible links between brain insulin signaling and cognitive ability.

## Introduction

Cognitive dysfunctions (CDs) feature among the major mental conditions threatening human health especially in older adults (Porter et al., 2019[66]). Cognitive dysfunction is described as "a significant impairment of cognition or memory that represents a marked deterioration from a previous level of function" (Cooper, 2018[16]; Shorter, 2022[74]). CDs negatively impact mental functioning and reduce or restrict the cognitive functions of learning, memory, perception and problem solving (Porter et al., 2019[66]; Viggiano et al., 2020[89]). They exhibit a wide array of mental symptoms including delirium, dementia, amnesia, anxiety and attention disorders (Cooper, 2018[16]). The latest edition of the Diagnostic and Statistical Manual of Mental Disorders (DSM), DSM-5-TR, featured a wide spectrum of mental disorders that are accompanied by varying levels of cognitive impairment (Bădescu et al., 2016[6]; Pinna et al., 2017[63]; Sastre et al., 2017[71]; Shorter, 2022[74]). Many studies have investigated the underlying causes of CDs and suggested that multiple pathophysiologic pathways are involved (Hampel et al., 2018[37]; Dewey et al., 2019[21]). Some of those studies have proposed a role for insulin, a peptide hormone primarily involved in metabolism and energy homeostasis, in cognition (Lv et al., 2020[54]; Barber et al., 2021[7]). Insulin can easily pass through the blood brain barrier (BBB) using a saturable transporter (Spinelli et al., 2019[77]). Insulin receptors (IRs) are widely expressed in different areas of the brain, including those related to cognitive performance (Hopkins and Williams 1997[41]; Spinelli et al., 2019[77]). Whilst insulin does not have a significant role in brain metabolic pathways and brain glucose homeostasis (Gabbouj et al., 2019[28]), it is involved in important activities such as neuronal growth, synaptic formation and plasticity (Gabbouj et al., 2019[28]). Altered levels of brain insulin resistance have been reported in patients with CDs (Hamer et al., 2019[36]; Spinelli et al., 2019[77]) and, therefore, this is highly suggestive of a relationship between cognitive ability and brain insulin signaling. In the current study, we discuss the potential extrametabolic roles of brain insulin signaling in cognitive functioning. 

## Cognitive Performance in Patients with Diabetes

Patients with uncontrolled diabetes mellitus (DM) often show cognitive impairments and memory loss such as Alzheimer's disease (AD), Parkinson's disease (PD) and dementia (Albai et al., 2019[2]; Chaytor et al., 2019[11]). An increased frequency of neuronal death and brain atrophy, due to increased apoptosis and necrosis, is the major pathological hallmark on brain magnetic resonance imaging (MRI) of patients with uncontrolled diabetes (Zilliox et al., 2016[99]; Moran et al., 2019[58]). Multiple pathophysiologic mechanisms are induced by DM and related dysfunctional metabolic pathways, including hexosamine, polyol and lipid peroxidation pathways, creating a toxic milieu around the neurons and ganglia involved in cognition and memory in the central and peripheral nervous systems. Thus, DM negatively impacts the normal physiological functioning of neuronal networks (Zilliox et al., 2016[99]). It has also been suggested that AD and DM have a shared pathophysiology that includes a distinct form of insulin resistance and impaired glucose tolerance in the brain and peripheral tissues (Sun et al., 2020[81]). Moreover, some recent reports have suggested a similar pathophysiology for DM and PD (Hassan et al., 2020[38]). Therefore, patients with diabetes are at increased risk of cognitive complications and memory loss relative to the non-diabetic population (Hogg et al., 2018[40]; Sang et al., 2018[70]).

## Insulin Signal Transduction

**Figure 1:**
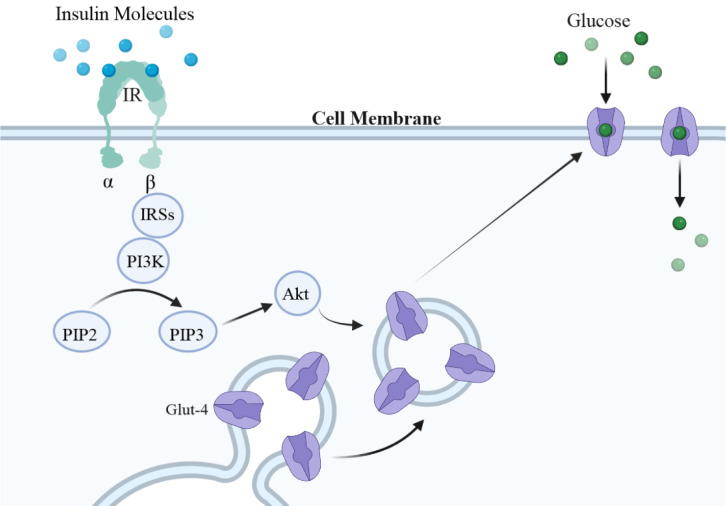
Insulin signaling machinery in peripheral tissues (IR= insulin receptor, IRS= insulin receptor substrate, PI3K=phosphoinositide 3-kinase, PIP_3_=phosphatidylinositol 3,4,5-trisphosphate, Akt= protein kinase b)

Insulin is a 51-amino acid peptide produced by pancreatic β-cells, mainly under the influence of circulating glucose, although other factors such as amino acids, acetylcholine, cholecystokinin and incretin hormones also play a role (Arnold et al., 2018[4]). Insulin signal transduction (IST) is initiated by binding of insulin to the α chain of its specific IR, a transmembrane tyrosine kinase composed of two chains, α and β (Færch et al., 2016[24]). This binding initiates β chain auto-phosphorylation in tyrosine residues followed by recruitment of adaptor proteins, insulin receptor substrates (IRSs), Shc protein (SHC-transforming protein), and APS protein (adapter protein with a PH and SH2 domain) (Kiselyov et al., 2009[45]; Hall, 2015[35]). These proteins provide an effective binding site for IRS-1 and cause its activation (Kiselyov et al., 2009[45]). Activated IRS-1 attaches to PI3K (phosphoinositide 3-kinase), activates it and catalyzes the conversion of PIP_2_ (Phosphatidylinositol 4,5-bisphosphate) to PIP_3_ (Phosphatidylinositol 3,4,5-trisphosphate) (Ho et al., 2016[39]). PIP_3 _is itself a potent activator for PKB (protein kinase B, also known as Akt) which, in turn, facilitates glucose entering into the cells by localization of Glut-4 (glucose transporter type 4) on the cell membrane of insulin-dependent tissues (Figure 1(Fig. 1)) (Ho et al., 2016[39]; Koeppen and Stanton, 2017[49]). 

Akt also inhibits glycogen synthase kinase (GSK) and induces glycogen synthesis (Ho et al., 2016[39]; Koeppen and Stanton 2017[49]). Several types of insulin-dependent kinases, such as ERK1/2 (extracellular signal‐regulated kinase 1/2), atypical PKC (protein kinase C), S6K1 (ribosomal protein S6 kinase beta-1), SIK2 (serine/threonine-protein kinase 2), AKT, mTOR (mammalian target of rapamycin) and ROCK1 (Rho-associated protein kinase 1) and other types of kinases such as AMPK (AMP-activated protein kinase) and GSK3 (Glycogen synthase kinase) can phosphorylate IRSs and activate them (Kiselyov et al., 2009[45]; Copps and White, 2012[17]).

## Brain Insulin Signaling

Brain tissue consumes about 20 % of all energy consumption in the body (Wardelmann et al., 2019[92]). The control of body energy homeostasis is mainly regulated by the brain and its own insulin/IGF-1 signaling to produce required energy, mainly by mitochondria in the form of ATP (Schell et al., 2021[73]). In addition to its metabolic role, it is now established that insulin (as well as insulin-like growth factor (IGF)) plays an important role as a neuromodulator (McNay and Recknagel 2011[56]). Brain insulin signaling is involved in control of body weight, food intake, reproduction, learning and memory (Kim and Feldman 2015[44]). Insulin promotes mitochondrial respiration and ATP production and modulates its dynamics (fission and fusion) in the brain (Schell et al., 2021[73]). Peripherally circulating insulin crosses the BBB via a saturable transport system, and enters the brain interstitial fluid (ISF) either directly through the BBB or via cerebrospinal fluid (CSF) (Mullins et al., 2017[59]). Also, whether or not *de novo* synthesis of insulin occurs in the brain is still debated (Kim and Feldman, 2015[44]). 

It has been established that IRs are extensively expressed in brain areas involved in memory and cognition such as the hippocampus (Zhao et al., 1999[96], 2004[97]), olfactory bulb, neocortex, cerebellum, hypothalamus (Choudhury et al., 2005[13]; Grillo et al., 2011[33]; Fernandez and Torres-Alemán, 2012[26]) and amygdala (Abbott et al., 1999[1]; Soto et al., 2019[76]). The pattern of IR expression is associated with behavioral activity and may be related to some cognitive disorders such as depression (Grillo et al., 2011[33]). The vast majority of IRs are localized on neurons, especially at synapses as a component of post-synaptic density (PSD) (Abbott et al., 1999[1]; Pomytkin and Pinelis, 2021[65]). Moreover, glucose transporters of Glut-4 are expressed in cerebellum, neocortex, astrocytes and the hippocampus, suggesting a role for insulin-dependent glucose uptake in neurons (Spinelli et al., 2019[77]). Furthermore, other molecules included in intracellular insulin signaling machinery, such as Akt, PI3K, mTOR, GSK3-β, CREB (transcription factors cAMP response element-binding protein) and FOXO (forkhead box O), are extensively present in neuronal tissues, evidence strongly suggestive of an important role in brain functioning (Fernandez and Torres-Alemán, 2012[26]; Kitagishi et al., 2012[46]; Inkster et al., 2018[42]; Rippin and Eldar-Finkelman, 2021[68]). 

## Possible Roles of Brain Insulin in Cognitive Functioning

Brain insulin is involved in many neuronal processes including dendritic sprouting, cell growth and repair, and neuronal stem cell activation (Stanciu et al., 2021[79]). Insulin exerts neuroprotective effects via control of phosphorylated tau levels and proinflammatory cytokines, which are both associated with β-amyloid (Aβ) depositions in the brain (Femminella et al., 2021[25]; Stanciu et al., 2021[79]). Emerging evidence suggests that cognitive disorders such as AD are naturally occurring metabolic disorders resulting from an inability to take up and utilize glucose (de la Monte, 2012[19]). Patients with diabetes commonly exhibit impaired brain insulin signaling which, in turn, facilitates cognitive deficit development (McNay and Recknagel, 2011[56]; Kim and Feldman, 2015[44]). Insulin resistance is also detected in the early stages of Down syndrome (Tramutola et al., 2020[86]). The expression level of genes involved in insulin signaling is reduced in patients with poor cognitive performance (Mullins et al., 2017[59]). Abnormal brain insulin signaling leads to alterations in many intracellular pathways, examples being decreased SREBP-2/SCAP (SREBP cleavage-activating protein)-dependent cholesterol synthesis, mitochondrial dysfunction, abnormal synaptic plasticity and increased levels of tau protein phosphorylation that, collectively, cause impaired neurological functioning and reduced cognitive abilities (Kleinridders et al., 2014[47]). 

In physiological conditions, insulin binding to its receptor at the synapse triggers IRS-1 phosphorylation following by PI3K-Akt pathway activation, GluA1 (Glutamate A1) phosphorylation and increased presence of GluN2B (Glutamate [NMDA] receptor subunit 2) at synapses which, in turn, favors synapse formation and memory function (Zilliox et al., 2016[99]). In the setting of diabetes with impaired brain insulin signaling, IR levels are reduced and GluN2B and GluA1 phosphorylation at synapses is also decreased (Zilliox et al., 2016[99]). Thus, synaptic plasticity and memory is impaired, while GSK-3b activity (which induces abnormal tau phosphorylation) is increased (Zilliox et al., 2016[99]). Moreover, brain insulin resistance is related to neurodegenerative processes, brain aging and poor cognitive abilities (Gorelick et al., 2011[32]; Spinelli et al., 2019[77]). Thus, dysregulated brain insulin or IGF signaling contributes to the cognitive decline induced by insulin resistance (Talbot et al., 2012[83]). In the following sections, we discuss and analyze the evidence regarding the possible roles of brain insulin signaling in cognitive functioning from a mechanistic viewpoint. 

### Brain insulin signaling and senile plaque formation

Senile plaques and neurofibrillary tangles are extracellular deposits of different subtypes of β-amyloid (Aβ) and tau proteins, and occur mainly in the grey matter of the brain cortex (Armstrong, 2009[3]). They are extracellular deposits of aberrantly processed, aggregated and misfolded oligomeric structural proteins, including Aβ peptides and hyperphosphorylated tau proteins, resulting from the abnormal processing of precursors by the β-and γ-secretase enzymes together with an imbalance between generation and clearance of Aβ peptides (de la Monte, 2012[19]; DeTure and Dickson, 2019[20]). Senile plaques are characterized by a central Aβ core surrounded by degenerated neurons in the extracellular space (Dickson, 1997[22]). These lesions are considered to be the principal histological hallmarks of neurodegeneration and AD (although they may also be seen in aging) and, as such, their frequency is directly associated with AD development and its dependent cognitive dysfunction (Dickson, 1997[22]; Armstrong, 2009[3]).

Available evidence suggests a close relationship between insulin signaling and senile plaque formation in the brain (Mullins et al., 2017[59]; Arvanitakis et al., 2020[5]; Ochiai et al., 2021[60]). Brain insulin resistance contributes to Aβ-dependent neurodegeneration and tau pathology, the main underlying features of AD (de la Monte, 2012[19]). Impaired brain insulin (or IGF) signaling leads to increased senile plaque formation through increased levels of Aβ and Aβ precursor protein (AβPP) expression and accumulation (Reich et al., 2018[67]). At the molecular level, insulin resistance and amyloidogenesis both interrupt common signaling pathways such as the IRs/PI3 kinase/Akt/GSK3 cascade and, therefore, they share overlapping pathology (Zhao and Townsend, 2009[98]; Kim and Feldman, 2015[44]). Reich and colleagues demonstrated that improvement in brain insulin sensitivity using PPAR (peroxisome proliferator activated receptor)-δ and PPAR-γ agonists decreases AβPP-Aβ accumulation and senile plaque formation in diabetic rats (Reich et al., 2018[67]). Similarly, Chua et al. demonstrated that impaired brain insulin signaling reduces glucose utilization and induces Aβ accumulation in the brain of female diabetic mice (Chua et al., 2012[14]). Another study demonstrated that high-fat diet (HFD)-induced insulin resistance accelerates Aβ accumulation and deposition in the brain of a mouse model of AD (Wakabayashi et al., 2019[91]). In a clinical study, Tramutola and coworkers found that increased insulin resistance was associated with Aβ accumulation in brain biopsies of Down syndrome patients (Tramutola et al., 2020[86]). 

Insulin inhibits Aβ intracellular accumulation and degradation via insulin-degrading enzyme (Gasparini et al., 2001[30], 2002[31]). In addition, brain insulin resistance promotes oxidative stress and alters energy homeostasis which, in turn, induces pro-AβPP-Aβ-mediated neurodegenerative cascades (de la Monte, 2012[19]). Moreover, senile plaques are able to intensify brain insulin resistance via different pathways such as autophagy-lysosomal- dependent insulin receptor degradation in BBB endothelial cells (Gali et al., 2019[29]). These findings highly suggest a close relationship between brain insulin signaling and Aβ and tau pathology (de la Monte, 2012[19]; Mullins et al., 2017[59]). Thus, brain insulin signaling contributes to cognitive efficiency through regulation of amyloid homeostasis and senile plaque formation (Wakabayashi et al., 2019[91]).

### Brain insulin signaling and neuro-synaptic plasticity

Synaptic plasticity, or modifications of synaptic transmission, has great impact on most neuronal processes including learning and memory (Citri and Malenka, 2008[15]). Normal neuronal plasticity, especially at synaptic junctions, helps to create more effective transmission and potentiates neuronal conduction, processes that play a significant role in memory acquisition and consolidation and cognitive function (Citri and Malenka, 2008[15]). Neuronal plasticity also has a major trophic role in uterine development of neural circuitry and enhances brain learning capacity and is therefore closely involved in both developing brain and adult brain functioning (Citri and Malenka, 2008[15]; Ferrario and Reagan, 2018[27]). Emerging evidence suggests that many cognitive disorders are associated with reduced synaptic plasticity (Lu et al., 2014[53]; Mayne and Burne, 2019[55]) and factors disrupting plasticity are able to reduce learning capacity and cause poor cognitive ability (Rogers et al., 2011[69]; Villeda et al., 2014[90]).

Insulin signaling has beneficial impacts on synaptic plasticity (Biessels et al., 1998[9]; Van Der Heide et al., 2005[88]; Spinelli et al., 2019[77]). It can exert both presynaptic and post-synaptic effects (Chiu and Cline, 2010[12]; Zhao et al., 2019[95]). Brain insulin resistance can suppress these processes and contribute to poor cognitive function via impairments in synaptic plasticity and neuroplasticity deficits (Stranahan et al., 2008[80]; Grillo et al., 2015[34]). The vast majority of IRs are localized on synaptic junctions as a component of PSD, implying that the synapse is an important site of brain insulin signaling (Abbott et al., 1999[1]; Pomytkin and Pinelis, 2021[65]). Insulin elicits memory (including food memory) in the hypothalamus (Choudhury et al., 2005[13]; Grillo et al., 2011[33]). It regulates hippocampal synaptic plasticity through several pathways such as NMDA (N‐methyl‐d‐aspartate) and PI3K dependent pathways (van der Heide et al., 2005[88]). Spatial memory training increases hippocampal insulin receptor expression, an area of brain where insulin receptors are present in higher concentrations than elsewhere (Zhao et al., 1999[96], 2004[97]). Similarly, an experimental model demonstrated that hippocampal insulin resistance was related to reduced synaptic plasticity as well as cognitive deficits in rats (Grillo et al., 2015[34]). Moreover, a genetic knockout of insulin receptors in the central nervous system (CNS) suppressed synaptic plasticity, reduced cognitive capacity and impaired hippocampal memory (Costello et al., 2012[18]). 

Insulin promotes hippocampal neuroplasticity by facilitating glutamatergic signaling and modifies mesolimbic circuits that mediate motivation and feeding behaviors (Ferrario and Reagan, 2018[27]). Insulin also regulates the VTA (ventral tegmental area)-NAc (nucleus accumbens) reward and motivation axis by modifying the synaptic plasticity in these circuits (Ferrario and Reagan, 2018[27]). It has been shown that local insulin injection in different brain areas modulates feeding and motivation behaviors, as well as cognitive functions, by increase of synaptic plasticity (Liu et al., 2013[52]; Tiedemann et al., 2017[84]; Zhao et al., 2019[95]). This effect was also observed in human studies in which intranasal insulin promoted memory (Benedict et al., 2004[8]). Insulin stimulated dendritic spine and excitatory synapse formation and induced LTP (long-term potentiation) via PI3K/Akt signaling pathways in the hippocampal area and thus modulated cognitive functions (Lee et al., 2011[51]; Zhao et al., 2019[95]). This trophic effect is dose-dependent and is associated with insulin signaling activity (Zhao et al., 2019[95]). Since hippocampus development continues even in adulthood (Braun and Jessberger, 2014[10]), brain insulin resistance in adults can suppress it and reduce cognitive function and memory efficiency (Arvanitakis et al., 2020[5]). Insulin modulates LTP and LTD (long-term depression) at hippocampal synapses (Spinelli et al., 2019[77]). Insulin decreases the stimulation frequency threshold required for inducing both LTP and LTD in hippocampal circuits (Spinelli et al., 2019[77]). 

Although glucose uptake is the main role of insulin throughout the body including in the brain, expression of insulin-independent glucose transporters (Glut-1, Glut-2, Glut-3, Glut-5, Glut-6, Glut-8 and Glut-13 that mediate glucose uptake into glial and neuronal cells) highly suggests that the impact of insulin on synaptic plasticity is independent of glucose uptake and is likely dependent upon insulin cascades such as the PI3K/Akt pathway (Joost and Thorens, 2001[43]; Membrez et al., 2006[57]; Simpson et al., 2007[75]; Spinelli et al., 2019[77]). Moreover, several growth factors, including IGF-1 and BDNF (brain-derived neurotrophic factor) that are involved in brain trophic pathways, are under the influence of the insulin cascade (Krabbe et al., 2007[50]; Dyer et al., 2016[23]). Taken together, it is apparent that insulin is a potent neurotrophic factor that is closely involved in neuro-synaptic plasticity and thus can promote synaptic transmission in brain areas related to cognitive ability (Zhao et al., 2019[95]). 

### Brain insulin signaling and mitochondrial integrity 

Mitochondria are known as the powerhouses of cells that provide required energy for cell survival and function; therefore, normal mitochondrial function and integrity is critically important for cells (Tzagoloff, 2012[87]). Mitochondrial dysfunction underlies the pathophysiology of both metabolic and non-metabolic complications as well as cognitive deficits (Pieczenik and Neustadt, 2007[62]; Otte et al., 2011[61]; de Filippis et al., 2015). Due to their high rate of metabolism, mitochondria are a major source of free radical production that can induce and promote redox imbalance and subsequent damage (Scaglia, 2010[72]; Otte et al., 2011[61]). Mitochondria-dependent oxidative stress is a main underlying cause of neuronal complications (Yaribeygi et al., 2018[94]). The CNS is highly dependent on oxidative phosphorylation that occurs in mitochondria and, therefore, mitochondrial insufficiency is usually accompanied by local brain necrosis (Leigh syndrome), static encephalopathy, dementia and cognitive deficits (Scaglia, 2010[72]). Many patients with mitochondrial encephalomyopathies (genetic impairment in mitochondrial energy production) have reduced levels of cognitive ability such as learning, memory, nonverbal cognitive impairment, compromised visuospatial abilities, perception and language (Scaglia, 2010[72]). Mitochondrial dysfunction may be linked to neuropsychiatric abnormalities such as dementia, depression, schizophrenia and bipolar disease (Scaglia, 2010[72]; Tobe, 2013[85]; Sripetchwandee et al., 2018[78]). Thus, normal mitochondrial function is required for CNS homeostasis and optimal cognitive ability. 

Recent evidence suggests that brain insulin signaling is closely associated with mitochondrial sufficiency (Wardelmann et al., 2019[92]; Pomytkin and Pinelis, 2021[65]). In addition, insulin resistance is associated with reduced mitochondrial respiration (Schell et al., 2021[73]). Wardelmann and colleagues found that brain insulin is an effective regulator of mitochondrial function (Wardelmann et al., 2019[92]); they demonstrated that intranasal insulin propagates mitochondrial function via up-regulation of genes involved in mitochondrial stress responses (Hsp60, Hsp10, Atf4, Chop, ClpP, and Lonp1) in both *in vitro* (hypothalamic cell line CLU183) and *in vivo* (high fat diet (HFD) fed mice) models and concluded that hypothalamic insulin signaling ensures mitochondrial function (Wardelmann et al., 2019[92]). Tramutola and coworkers found that brain insulin resistance, as observed in patients with Down syndrome, is associated with mitochondrial dysfunction (Tramutola et al., 2020[86]); they reported that abnormal brain insulin signaling in these patients is directly linked to reduced expression of proteins involved in mitochondrial complexes II, III and IV (Tramutola et al., 2020[86]).

Insulin resistance is associated with mitochondrial deficits in a female AD mouse model (Yao et al., 2009[93]). Improving brain insulin signaling using rosiglitazone (a peroxisome proliferator-activated receptor γ agonist) improves brain mitochondrial efficiency (Pipatpiboon et al., 2012[64]). Pipatpiboon et al. demonstrated that rosiglitazone not only increases brain insulin sensitivity, but also improves mitochondrial function and cognitive performance in HFD rats (Pipatpiboon et al., 2012[64]). Recent findings indicate that people with PD have dysregulated forms of insulin-dependent mitochondrial chaperone Hsp10 in their brain tissue (Szegő et al., 2019[82]). Additionally, other roles have been suggested for insulin-dependent mitochondrial integrity in the brain, such as control of ion homeostasis, neuroapoptosis, endoplasmic reticulum (ER) stress and autophagy (Schell et al., 2021[73]). Therefore, it seems that there is important crosstalk between brain insulin signaling and mitochondrial integrity (Kleinridders et al., 2018[48]); this link ensures neuronal respiration and energy balance that are necessary for proper neuronal functioning and cognitive ability (Kleinridders et al., 2018[48]; Schell et al., 2021[73]). 

## Conclusion

**Figure 2:**
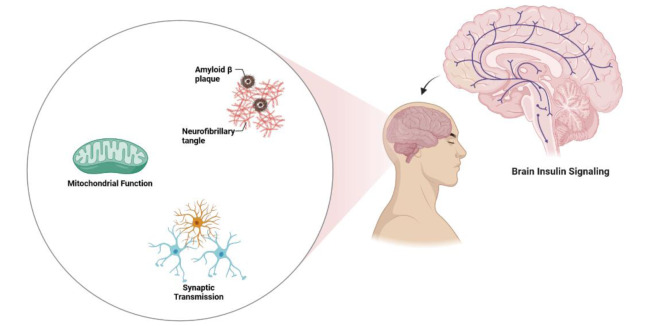
Brain insulin signaling is involved in cognitive function via at least three pathways: modulating senile plaque formation, synaptic transmission and mitochondrial efficiency in the brain.

Insulin signaling has critical extra-metabolic roles in the brain. It is involved in many neuronal and neurobehavioral processes and induces and promotes neurogenesis even in adulthood. Recent evidence suggests that insulin signaling also has a prominent role in cognition and memory, and people with poor cognitive ability have impaired brain insulin signaling. Experimental and clinical studies using interventions modulating brain insulin signaling have the potential to effectively modify cognitive capacity by yet poorly defined mechanisms. Our current analysis proposes that proper brain insulin signaling is involved in normal cognitive ability via at least three different mechanisms: (1) preventing or reducing senile plaque formation in brain areas related to cognition and memory, (2) inducing and promoting neurosynaptic plasticity and potentiating synaptic transmission, and (3) maintaining mitochondrial integrity in brain tissue (Figure 2(Fig. 2)). These mechanisms are crucial for proper cognitive functioning and prevention of neurodegenerative disorders and are influenced by brain insulin signaling (Table 1(Tab. 1); References in Table 1: Arvanitakis et al., 2020[5]; Lee et al., 2011[51]; Mullins et al., 2017[59]; Ochiai et al., 2021[60]; Schell et al., 2021[73]; Spinelli et al., 2019[77]; Szegő et al., 2019[82]; Wardelmann et al., 2019[92]; Yao et al., 2009[93]; Zhao et al., 2019[95]).

## Notes

Habib Yaribeygi and Amirhossein Sahebkar (Applied Biomedical Research Center, Mashhad University of Medical Sciences, Mashhad, Iran, E-mail: amir_saheb2000@yahoo.com) contributed equally as corresponding author.

## Conflict of interest

The authors clearly declare that have no conflict of interest in this study. 

## Figures and Tables

**Table 1:**
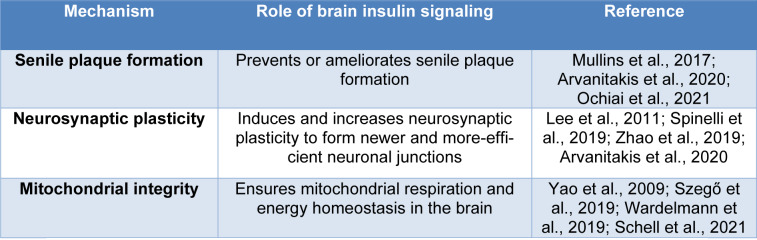
Brain insulin signaling modulates cognitive ability via 3 distinct mechanisms.
